# Supplementary material for: Single-cell transcriptional networks in differentiating preadipocytes suggest drivers associated with tissue heterogeneity
Source: Nat Commun. 2020 Apr 30;11:2117. doi: 10.1038/s41467-020-16019-9 (PMC7192917; doi:10.1038/s41467-020-16019-9)
Supplement: Supplementary file 1 — Supplementary Information [file 41467_2020_16019_MOESM1_ESM.pdf]

Supplementary Information

Ramirez et al. Single-Cell Transcriptional Networks in Differentiating Preadipocytes Suggest Drivers Associated with Tissue Heterogeneity

This file includes Supplementary Figures 1-9 and Supplementary Methods.

Supplementary Figures

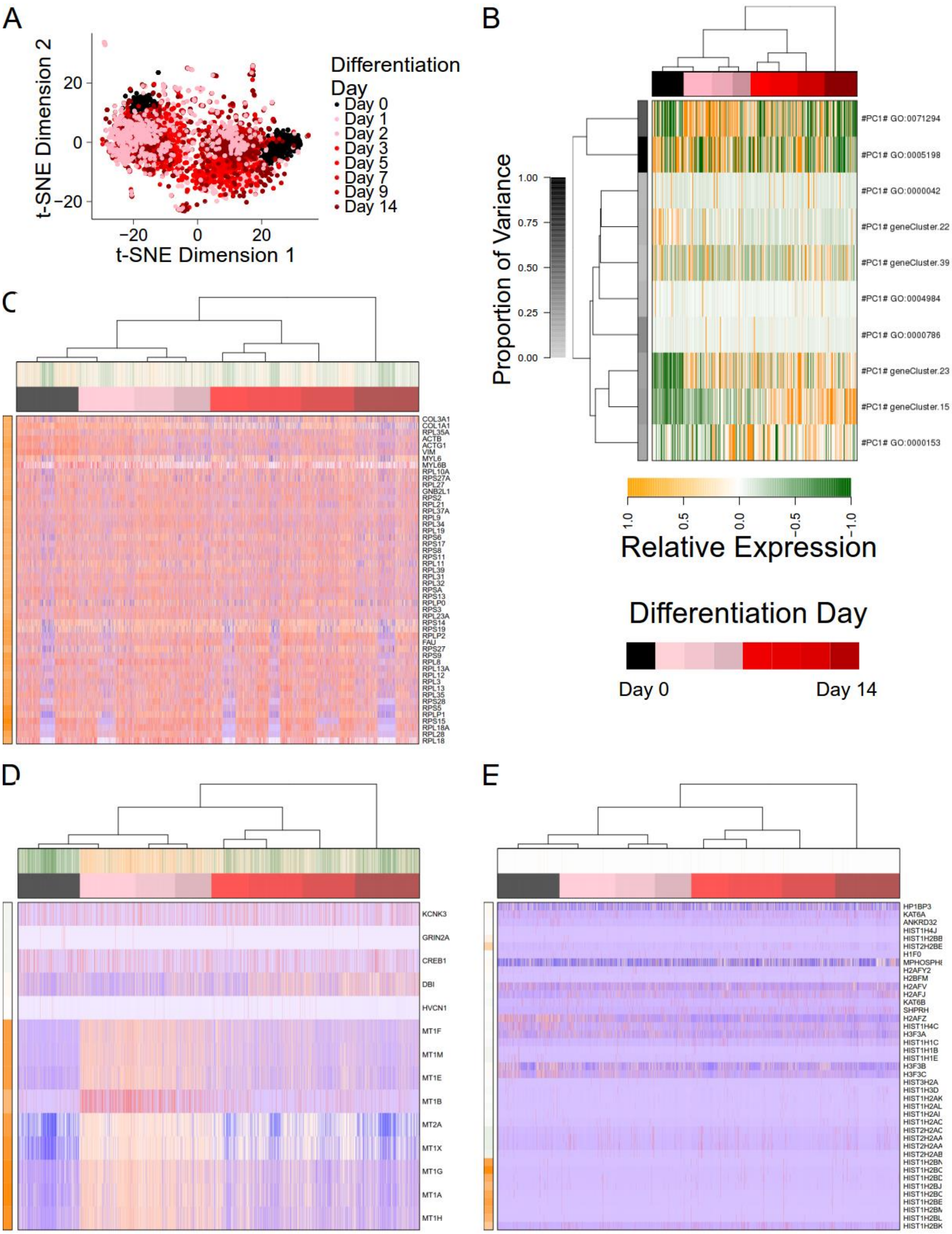

Supplementary Figure 1 PAGODA and t-SNE reveals at least two clusters in differentiating preadipocytes. Single-cell RNA seq was performed on differentiating preadipocytes beginning at 100% confluency and differentiated for 14 days. PAGODA was used to determine the optimal cell clustering based on the genes driving the heterogeneity. (A) Differentiating preadipocytes beginning at 80% show two distinct clusters of cells most evident at day 0. (B) The heatmap of the significance gene sets shows a few de novo gene sets captured major aspects of heterogeneity. (C) The highest-ranking gene set had the highest-expressing cells covering all days of differentiation. (D) The second highest-ranking gene set had the highest-expressing cells in the intermediate days of differentiation (days 3, 5, 7). (E) The third highest-ranking gene set had the highest-expressing cells in early days of differentiation (days 0, 1, and 2).

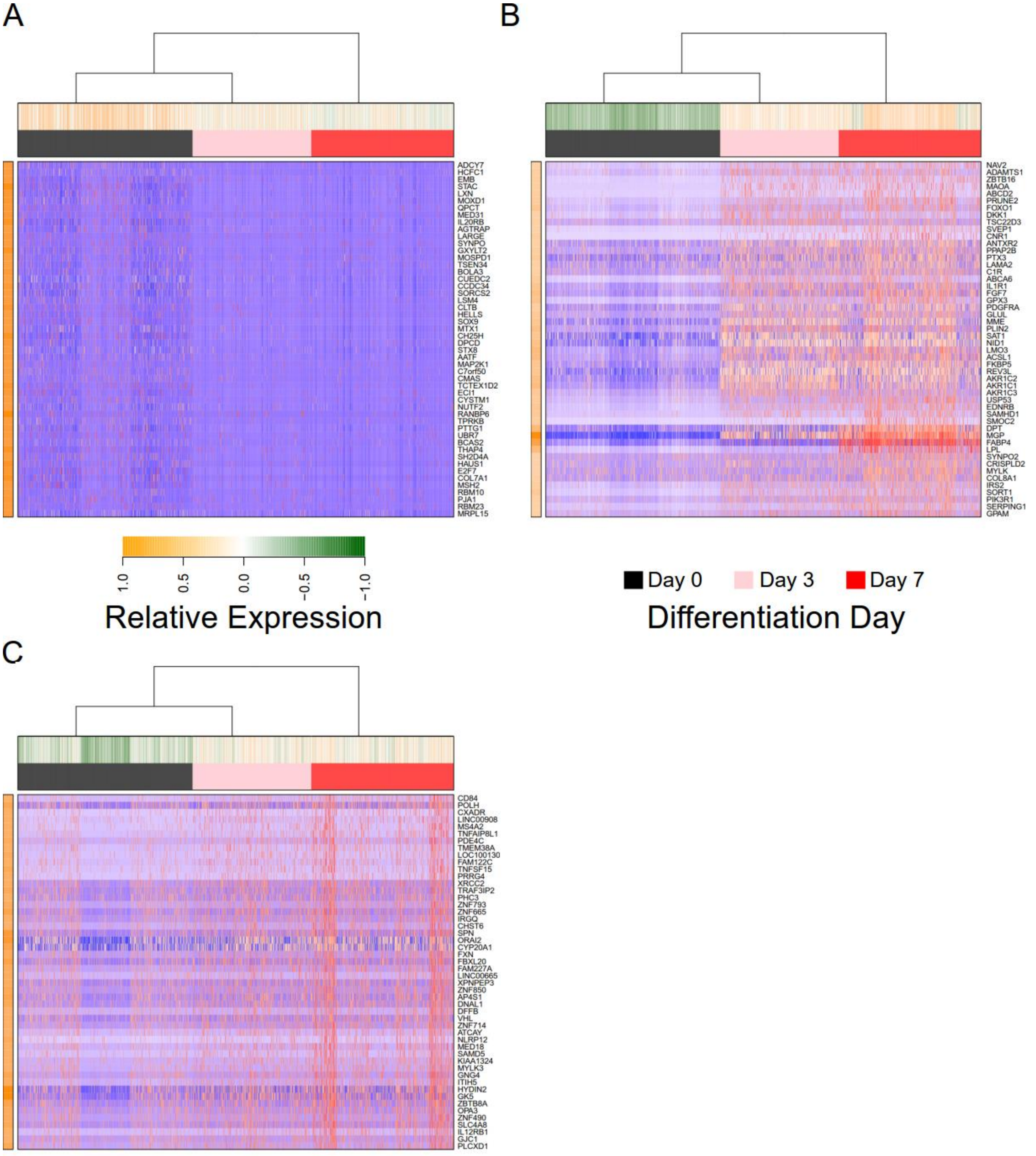
A
B
C
ADCY7
HCFC1
EMB
STAC
LXN
MOXD1
QPCT
MED31
IL20RB
AGTRAP
LARGE
SYNPO
GXYLT2
MOSPD1
TSEN34
BOLA3
CUEDC2
CCDC34
SORCS2
LSM4
CLTB
HELLS
SOX9
MTX1
CH25H
DPCD
STX8
AATF
MAP2K1
C7orf50
CMAS
TCTEX1D2
ECI1
CYSTM1
NUTF2
RANBP6
TPRKB
PTTG1
UBR7
BCAS2
THAP4
SH2D4A
HAUS1
E2F7
COL7A1
MSH2
RBM10
PJA1
RBM23
MRPL15
NAV2
ADAMTS1
ZBTB16
MAOA
ABCD2
PRUNE2
FOXO1
DKK1
TSC22D3
SVEP1
CNR1
ANTXR2
PPAP2B
PTX3
LAMA2
C1R
ABCA6
IL1R1
FGF7
GPX3
PDGFRA
GLUL
MME
PLIN2
SAT1
NID1
LMO3
ACSL1
FKBP5
REV3L
AKR1C2
AKR1C1
AKR1C3
USP53
EDNRB
SAMHD1
SMOC2
DPT
MGP
FABP4
LPL
SYNPO2
CRISPLD2
MYLK
COL8A1
IRS2
SORT1
PIK3R1
SERPING1
GPAM
1.0
0.5
0.0
−0.5
−1.0
Relative Expression
Day 0
Day 3
Day 7
Differentiation Day
CD84
POLH
CXADR
LINC00908
MS4A2
TNFAIP8L1
PDE4C
TMEM38A
LOC100130
FAM122C
TNFSF15
PRRG4
XRCC2
TRAF3IP2
PHC3
ZNF793
ZNF665
IRGQ
CHST6
SPN
ORAI2
CYP20A1
FXN
FBXL20
FAM227A
LINC00665
XPNPEP3
ZNF850
AP4S1
DNAL1
DFFB
VHL
ZNF714
ATCAY
NLRP12
MED18
SAMD5
KIAA1324
MYLK3
GNG4
ITIH5
HYDIN2
GK5
ZBTB8A
OPA3
ZNF490
SLC4A8
IL12RB1
GJC1
PLCXD1

Supplementary Figure 2 PAGODA suggests a few significant gene sets drive the transcriptional heterogeneity in differentiating preadipocytes beginning at 100% confluency. (A) The highest-ranking gene set had the highest-expressing cells in several day 0 preadipocytes. (B) The second highest-ranking gene set had the highest-expressing cells in day 3 and day 7 differentiating preadipocytes. (C) The third highest-ranking gene set had the highest-expressing cells in day 7 adipocytes.

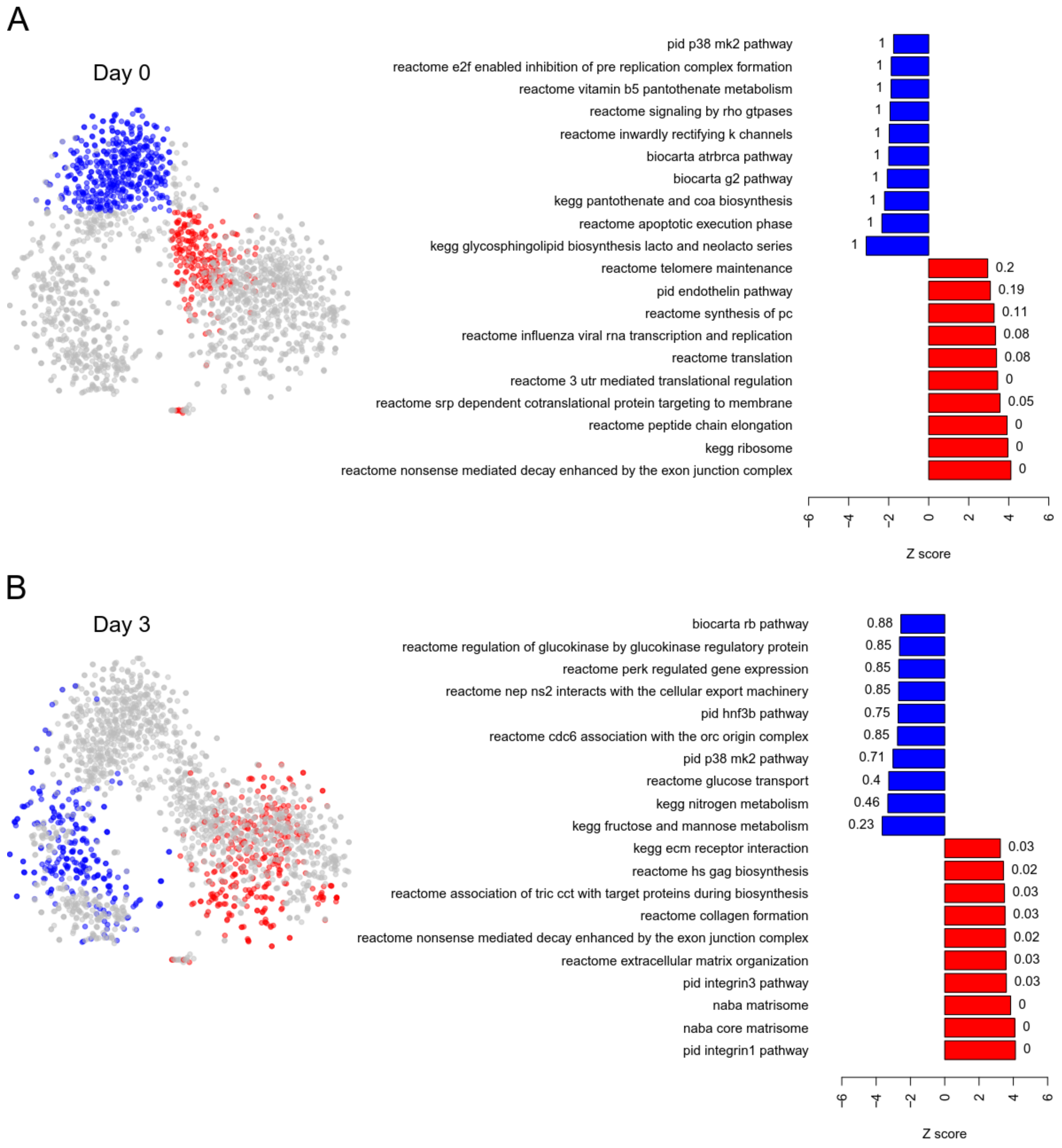

A
Day 0
pid p38 mk2 pathway
reactome e2f enabled inhibition of pre replication complex formation
reactome vitamin b5 pantothenate metabolism
reactome signaling by rho gtpases
reactome inwardly rectifying k channels
biocarta atrbrca pathway
biocarta g2 pathway
kegg pantothenate and coa biosynthesis
reactome apoptotic execution phase
kegg glycosphingolipid biosynthesis lacto and neolacto series
reactome telomere maintenance
pid endothelin pathway
reactome synthesis of pc
reactome influenza viral rna transcription and replication
reactome translation
reactome 3 utr mediated translational regulation
reactome srp dependent cotranslational protein targeting to membrane
reactome peptide chain elongation
kegg ribosome
reactome nonsense mediated decay enhanced by the exon junction complex
1
1
1
1
1
1
1
1
1
1
0.2
0.19
0.11
0.08
0.08
0
0.05
0
0
0
-6 -4 -2 0 2 4 6
Z score
B
Day 3
biocarta rb pathway
reactome regulation of glucokinase by glucokinase regulatory protein
reactome perk regulated gene expression
reactome nep ns2 interacts with the cellular export machinery
pid hnf3b pathway
reactome cdc6 association with the orc origin complex
pid p38 mk2 pathway
reactome glucose transport
kegg nitrogen metabolism
kegg fructose and mannose metabolism
kegg ecm receptor interaction
reactome hs gag biosynthesis
reactome association of tric cct with target proteins during biosynthesis
reactome collagen formation
reactome nonsense mediated decay enhanced by the exon junction complex
reactome extracellular matrix organization
pid integrin3 pathway
naba matrisome
naba core matrisome
pid integrin1 pathway
0.88
0.85
0.85
0.85
0.75
0.85
0.71
0.4
0.46
0.23
0.03
0.02
0.03
0.03
0.02
0.03
0.03
0
0
0
-6 -4 -2 0 2 4 6
Z score

Supplementary Figure 3 Differential gene expression shows day-specific enrichment of pathways between the two clusters of differentiating preadipocytes. Differential gene expression was performed between the left (blue) and right (red) cluster for each day of differentiation in differentiating preadipocytes beginning at 100% confluency. Gene set enrichment analysis was performed on the differentially expressed genes and the top 10 up- and down-regulated pathways sorted by z-score are shown for each day. The numbers next to the bars indicate the false discovery rate (FDR). (A) At day 0, the genes in the right cluster were related to several gene sets involving protein translation, whereas the left cluster had no significant gene sets (FDR < 0.25). (B) At day 3, the right cluster had several gene sets related to extracellular matrix remodeling, whereas the left cluster had one significant gene set in fructose metabolism.

A

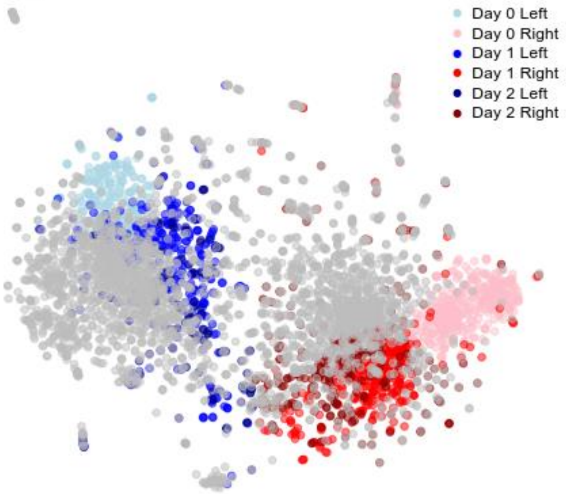

	Day of Differentiation					
	0	1	2	0	1	2
Reactome GPCR Ligand Binding	1	1	0.99	0	0.12	0.98
Reactome Class A1 Rhodopsin-like Receptors	1	1	0.99	0	0.12	0.98
Kegg Neuroactive Ligand Receptor Interaction	1	1	0.99	0	0	0.98
Reactome Generic Transcription Pathway	1	1	0.97	0	0	0.98
Kegg Cytokine Cytokine Receptor Interaction	1	1	1	0	0.12	0.88
Reactome TCA Cycle and Respiratory Electron Transport	0	0	0.97	1	1	0.98
Kegg Alzheimers Disease	0	0	0.97	1	1	0.98
Reactome Activation of the mRNA Upon Binding...	0	0	0.97	1	1	0.98
Reactome Metabolism of RNA	0	0	0.97	1	1	0.98
Reactome HIV Infection	0	0	0.97	1	1	0.98

B

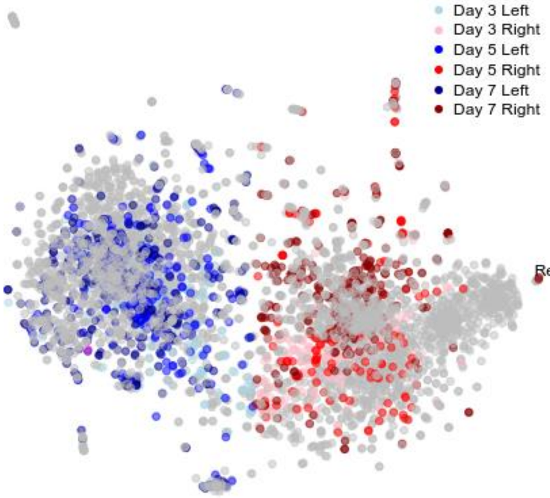

	Day of Differentiation					
	3	5	7	3	5	7
Reactome Translation	1	1	1	0	0.98	0
Reactome Influenza Life Cycle	1	1	1	0	0.98	0
Reactome 3' Mediated Transcriptional Regulation	1	1	1	0	0.98	0
Reactome SRP Dependent Protein Targeting to Membrane	1	1	1	0	0.98	0
Reactome Formation of Ternary Complex and the 43S Complex	1	1	1	0	0.99	0
Reactome Metabolism of Proteins	1	1	1	0	0.99	0
Reactome Peptide Chain Elongation	1	1	1	0	0.95	0
Kegg Ribosome	1	1	1	0	0.94	0
Reactome Influenza Viral RNA Transcription and Replication	1	1	1	0	0.96	0
Reactome Metabolism of mRNA	1	1	1	0	0.89	0

C

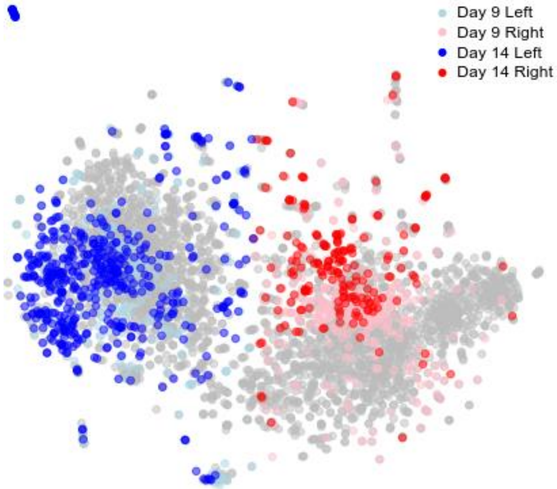

	Day of Differentiation			
	9	14	9	14
Kegg Glycolysis Gluconeogenesis	0.33	0.056	1	1
Reactome Respiratory Electron Transport	0.29	0	1	1
Kegg Alzheimers Disease	0.27	0	1	1
Kegg Parkinsons Disease	0.33	0	1	1
Kegg Oxidative Phosphorylation	0.32	0	1	1
Kegg Huntingtons Disease	0.13	0.019	1	1
Reactome Glucose Metabolism	0.25	0.33	1	1
PID Insulin Pathway	0.11	0.31	1	1
Reactome Prolonged ERK Activation Events	0.31	1	1	0.23
PID Telomerase Pathway	0.14	1	1	0

Supplementary Figure 4 Differential gene expression shows stage-specific enrichment of pathways between the two clusters of differentiating preadipocytes. Differential gene expression was performed between the left (shades of blue) and right (shades of red) clusters for each day of differentiation in differentiating preadipocytes beginning at 100% confluency. Gene set enrichment analysis was performed on the differentially expressed genes and summarized representative five up- and down-regulated pathways for three majors stages of differentiation were shown. The numbers in the heatmap indicate the false discovery rate (FDR). (A) At the early stages of differentiation (days 0, 1, and 2), the right clusters were enriched in pathways related to G-protein coupled receptor binding. The left clusters were enriched in pathways related to RNA metabolism. (B) At the intermediate stages of differentiation (days 3, 5, and 7), the right clusters were enriched in pathways related to protein synthesis. The left clusters had no enriched pathways (FDR <0.25). (C) In the late stages of differentiation (days 9 and 14), the right clusters were enriched in the telomerase pathway. The left clusters were enriched in several metabolic gene sets including glycolysis, respiratory electron transport, and glucose metabolism.

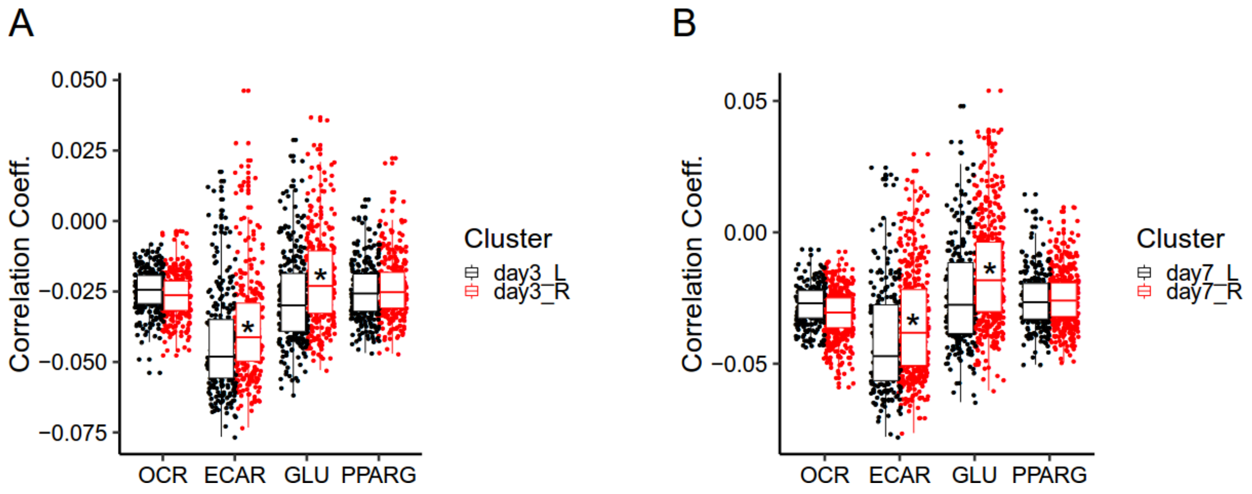

Supplementary Figure 5 Single cells during adipogenesis marked by genes correlated to extracellular acidification and glucose uptake in clonal preadipocyte cell lines. RNA-seq was performed on 36 clonally-expanded white preadipocyte cell lines derived from human subcutaneous neck white adipose tissue. These gene expression profiles were correlated (Pearson) to measured phenotypes in Figure 2. The four phenotype correlation vectors (oxygen consumption, extracellular acidification, glucose uptake, and PPARG expression after differentiation) were then correlated (Spearman) to the single-cell gene expression profiles for each cluster of cells differentiated at 100% confluency at day 3 (A) or day 7 (B). The distribution of correlation coefficients was compared between the left and right cluster. The left and right clusters show significant differences in extracellular acidification and glucose uptake. Boxplots show median and the interquartile range per cell cluster. Points indicate correlation coefficients of individual cells. Asterisks indicate FWER < 0.05 as assessed by a one-way ANOVA followed by a Bonferroni correction. Boxplots are centered on the median, the interquartile range (IQR) spans the 25-75% percentile, and the whiskers extend to 1.5 times the IQR above the 75% percentile (maximum) and below the 25% percentile (minimum).

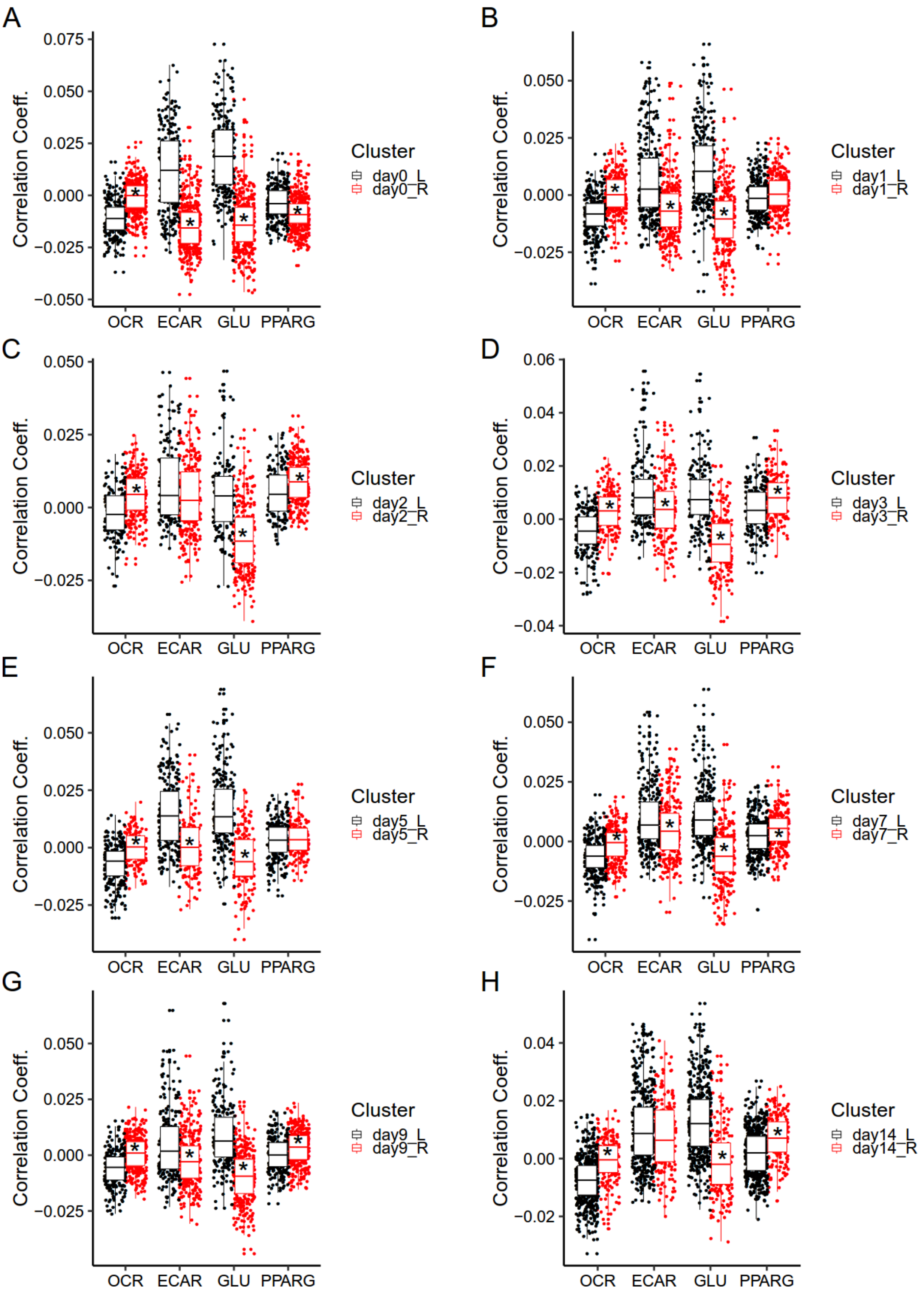

A
Correlation Coeff.
0.075
0.050
0.025
0.000
−0.025
−0.050
OCR
ECAR
GLU
PPARG
Cluster
day0_L
day0_R
B
Correlation Coeff.
0.050
0.025
0.000
−0.025
Cluster
day1_L
day1_R
C
Correlation Coeff.
Cluster
day2_L
day2_R
D
Correlation Coeff.
0.06
0.04
0.02
0.00
−0.02
−0.04
Cluster
day3_L
day3_R
E
Correlation Coeff.
Cluster
day5_L
day5_R
F
Correlation Coeff.
Cluster
day7_L
day7_R
G
Correlation Coeff.
Cluster
day9_L
day9_R
H
Correlation Coeff.
Cluster
day14_L
day14_R

Supplementary Figure 6 Single cells during adipogenesis marked by genes correlated to extracellular acidification and glucose uptake in clonal preadipocyte cell lines. RNA-seq was performed on 36 clonally-expanded white preadipocyte cell lines derived from human subcutaneous neck white adipose tissue. These gene expression profiles were correlated (Pearson) to measured phenotypes in Figure 2. The four phenotype correlation vectors (oxygen consumption, extracellular acidification, glucose uptake, and PPARG expression after differentiation) were then correlated (Spearman) to the single-cell gene expression profiles for each cluster of the cells differentiated at 100% confluency. The distribution of correlation coefficients was compared between the left and right cluster for each day. In each day (A-H), the left and right clusters show significant differences in extracellular acidification and glucose uptake. Boxplots show median and the interquartile range per cell cluster. Points indicate correlation coefficients of individual cells. Asterisks indicate FWER < 0.05 as assessed by a one-way ANOVA followed by a Bonferroni correction. Boxplots are centered on the median, the interquartile range (IQR) spans the 25-75% percentile, and the whiskers extend to 1.5 times the IQR above the 75% percentile (maximum) and below the 25% percentile (minimum).

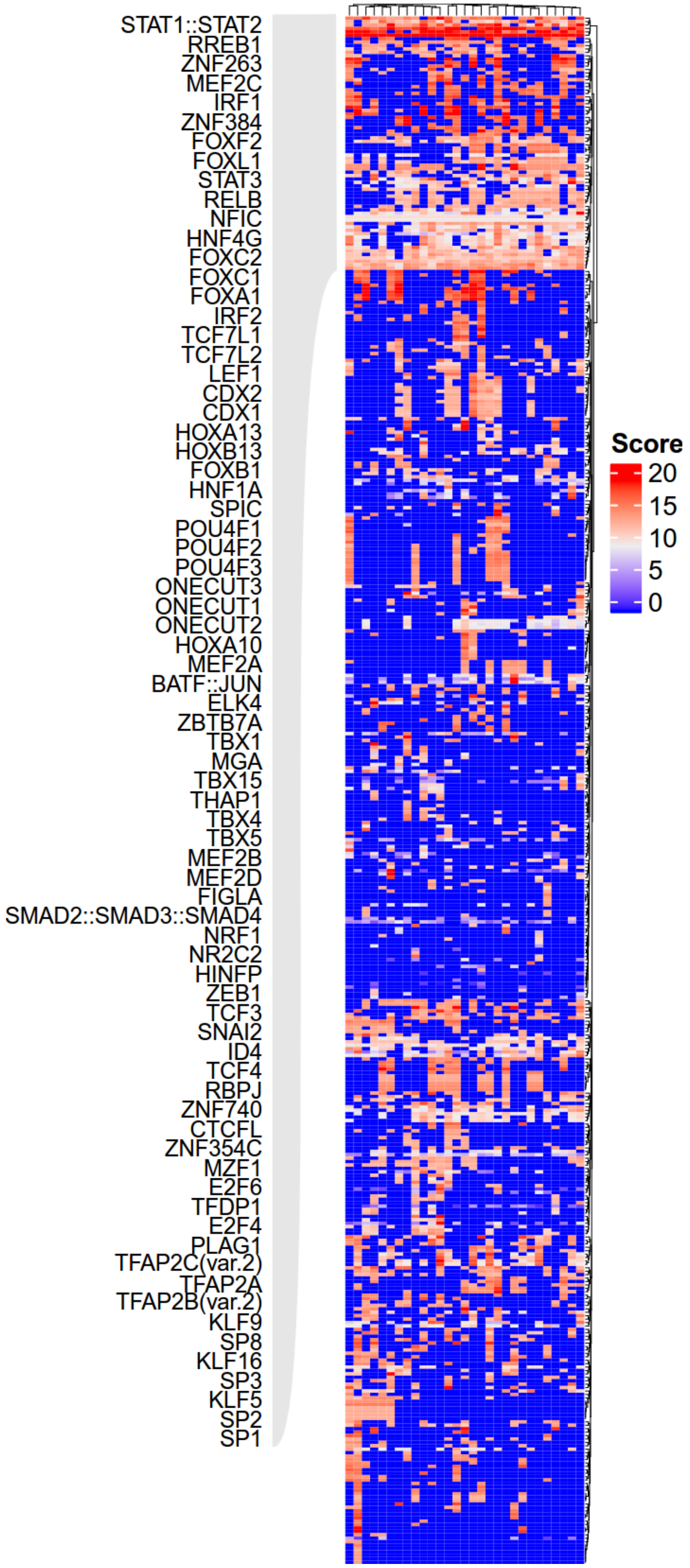
STAT1::STAT2
RREB1
ZNF263
MEF2C
IRF1
ZNF384
FOXF2
FOXL1
STAT3
RELB
NFIC
HNF4G
FOXC2
FOXC1
FOXA1
IRF2
TCF7L1
TCF7L2
LEF1
CDX2
CDX1
HOXA13
HOXB13
FOXB1
HNF1A
SPIC
POU4F1
POU4F2
POU4F3
ONECUT3
ONECUT1
ONECUT2
HOXA10
MEF2A
BATF::JUN
ELK4
ZBTB7A
TBX1
MGA
TBX15
THAP1
TBX4
TBX5
MEF2B
MEF2D
FIGLA
SMAD2::SMAD3::SMAD4
NRF1
NR2C2
HINFP
ZEB1
TCF3
SNAI2
ID4
TCF4
RBPJ
ZNF740
CTCFL
ZNF354C
MZF1
E2F6
TFDP1
E2F4
PLAG1
TFAP2C(var.2)
TFAP2A
TFAP2B(var.2)
KLF9
SP8
KLF16
SP3
KLF5
SP2
SP1
Score
20
15
10
5
0

Supplementary Figure 7 Motif analysis of ZNF subnetwork promoter regions. A motif search was performed using the promoters of each gene in the 30-genes network against all the motifs provided in the JASPAR 2018 database. A heatmap showing the alignment scores of the search was plotted for every transcription factor.

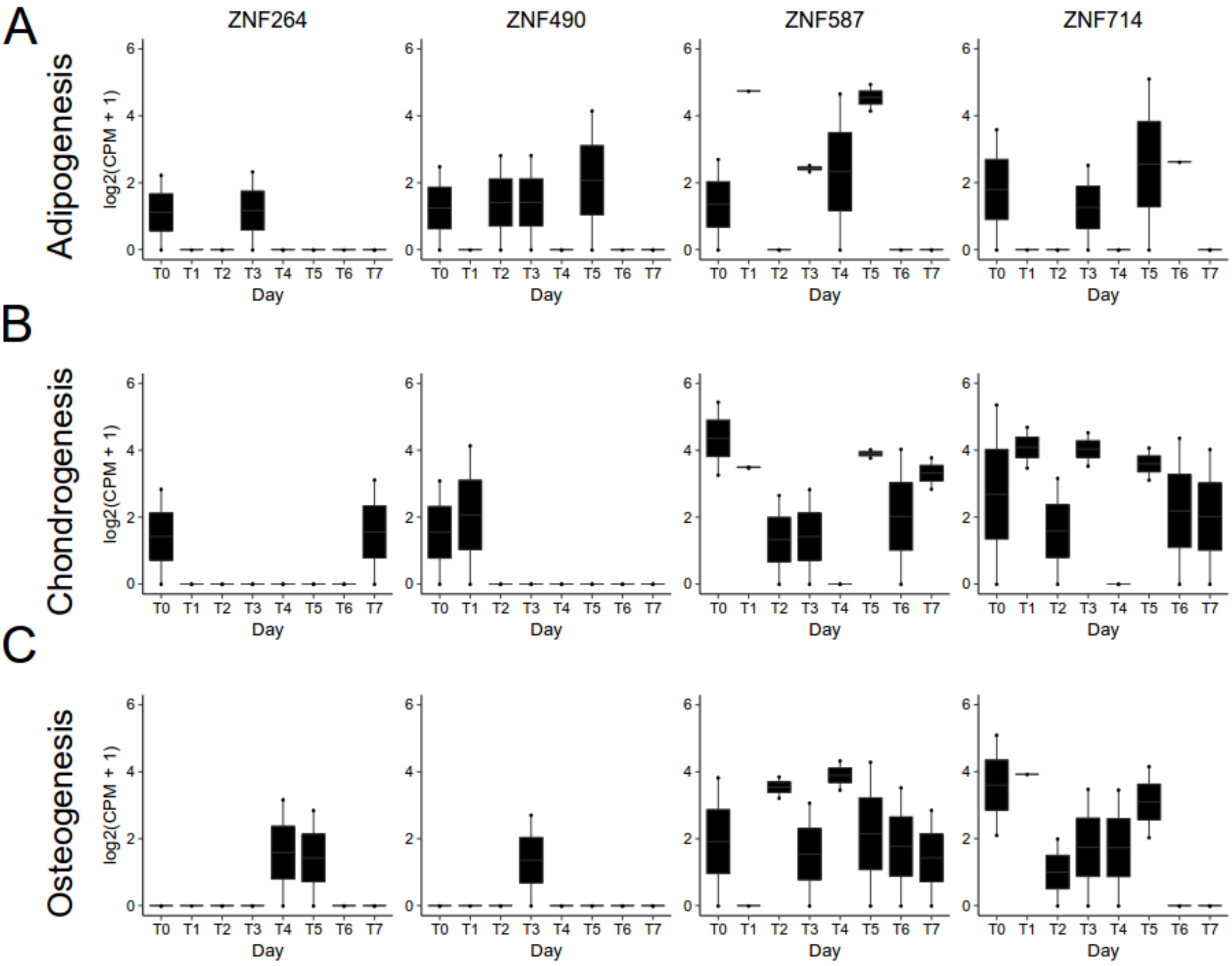

Supplementary Figure 8 ZNFs may be concordantly downregulated in adipogenesis, but not in chondrogenesis or osteogenesis. RNA-seq expression profiles generated during the differentiation of fibroblasts into adipocytes, chondrocytes, or osteocytes were obtained from GEO GSE37521 (see Methods). The expression of ZNF264, ZNF490, ZNF587, and ZNF714 show lineage-specific regulation during differentiation. (A) Adipogenesis shows higher expression of the ZNF cluster in the undifferentiated state (day 0) compared to day 7 (T7). (B) Chondrogenesis suggests a time-dependent decline in ZNF490 but not other ZNFs. (C) Osteogenesis does not show time-dependent regulation of the ZNFs. Bars indicate mean ± s.e.m. N = 2 people. Boxplots are centered on the median, the interquartile range (IQR) spans the 25-75%

percentile, and the whiskers extend to 1.5 times the IQR above the 75% percentile (maximum) and below the 25% percentile (minimum).

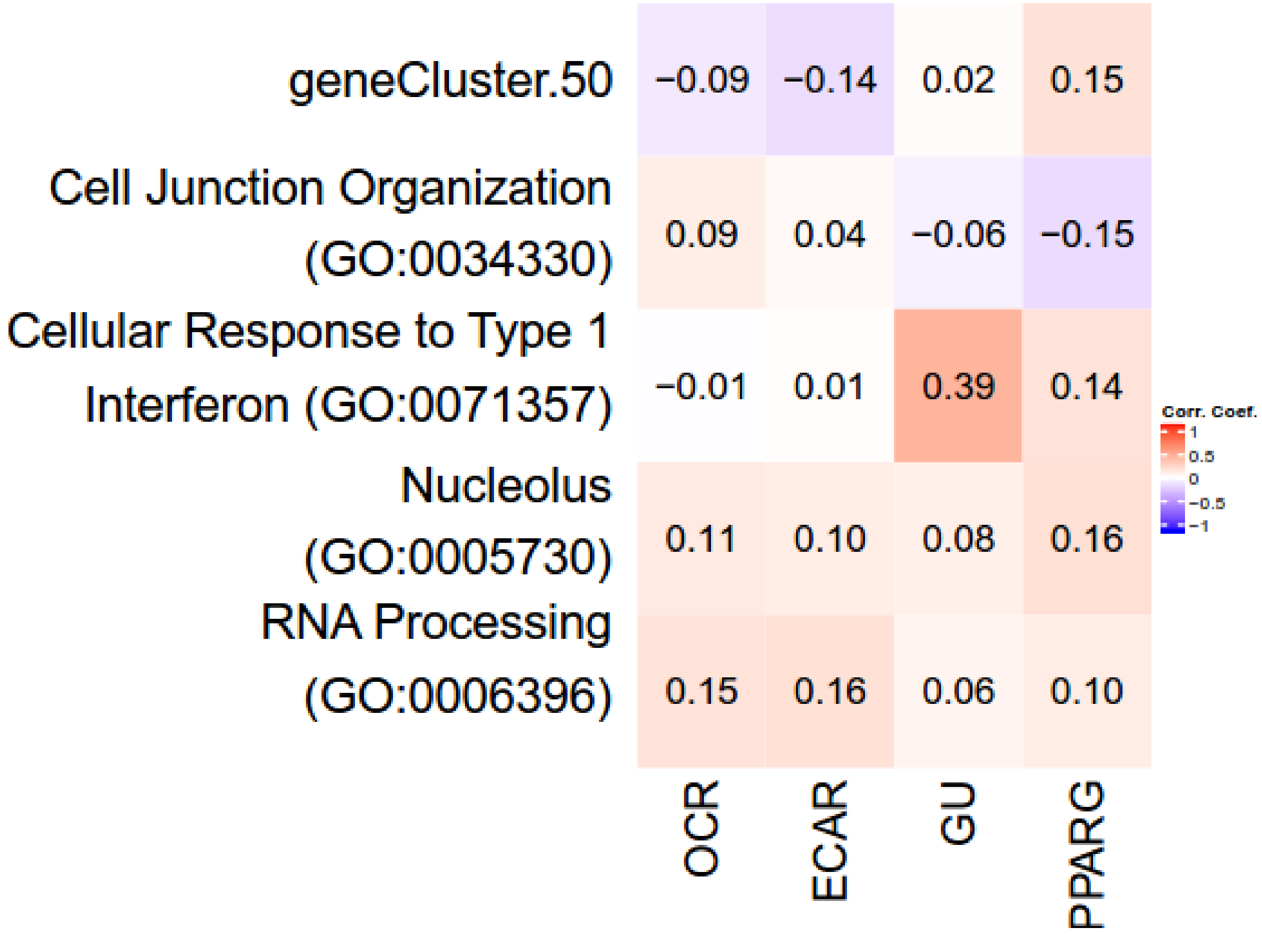

Supplementary Figure 9 A total of four phenotypes were correlated (Pearson) to the five significant aspects. Values indicate correlation coefficient (N=35 different cells lines after quality control). OCR = Oxygen Consumption Rate. ECAR = Extracellular Acidification Rate. GU = Glucose Uptake. PPARG = PPARG after differentiation.

Supplementary Methods

A Network Decomposition Algorithm for Single-cell RNA Sequencing

In this section, we describe an algorithm for detecting active connected subnetworks in the PPI network such that the observed expression of each gene across all cells is regulated (and approximated) by the sum the activities of subnetworks in which the gene is a member.

The inputs to the algorithm are an expression matrix $X \in \mathbb{R}^{m \times n}$ and a PPI network $M(V, E)$. The rows in the expression matrix correspond to samples or cells and the columns correspond to genes. Each node in M is associated with a column (gene) in X.

The PPI network is obtained from Pathway Commons.

Moreover, the algorithm has 3 input parameters: the number of subnetworks to be found (denoted by r), the maximum size of any subnetwork (denoted by l), and an integer s that specifies the number of seed nodes used in the algorithm as described below. The parameter *r* roughly corresponds to the more traditional number of gene clusters in gene expression data we are seeking to identify.

The output of the algorithm consists of detected subnetworks in the PPI network along with associated cell-specific activity levels for each subnetwork. For a given number of subnetworks r and for $k \in \{1, \ldots, r\}$, we denote the kth subnetwork with a binary indicator vector g_k of length n. We denote the activity level of subnetwork k over cells with a real vector c_k of length m. The algorithm generates an approximation for the input matrix that can be expressed as: $\tilde{X} = \sum_{i=1}^{r} c_i\, g_i^T$

More compactly, we collect $g_1, \cdots, g_r$ into a matrix G such that $G_{ij} = 1$ iff network *i* includes gene *j.* Then we can write the problem that our algorithm is trying to solve as:

$$\min_{C,G} ||X - CG^T||_F^2$$

where each row in $G^T \in \{0,1\}^{r \times n}$ denotes a *connected* subnetwork whose signature activity over cells forms the corresponding column of $C \in \mathbb{R}^{m \times n}$. Supplementary Figure 10 shows an example of the network decomposition for $r = 2$.

We develop a heuristic algorithm to solve the above problem. Our algorithm consists of two simpler procedures:

1) a greedy method that consecutively finds connected subnetworks, such that each solution best approximates the residual data matrix that is obtained after subtracting previous solutions.

2) a greedy method to find a single connected subnetwork g and a signature vector c for a given residual data matrix X_{res} such that assigning the signature vector to all the genes in the detected subnetwork minimizes the reconstruction error of the residual matrix, i.e., $\min_{c,g} ||X_{res} - cg^T||_F^2$.

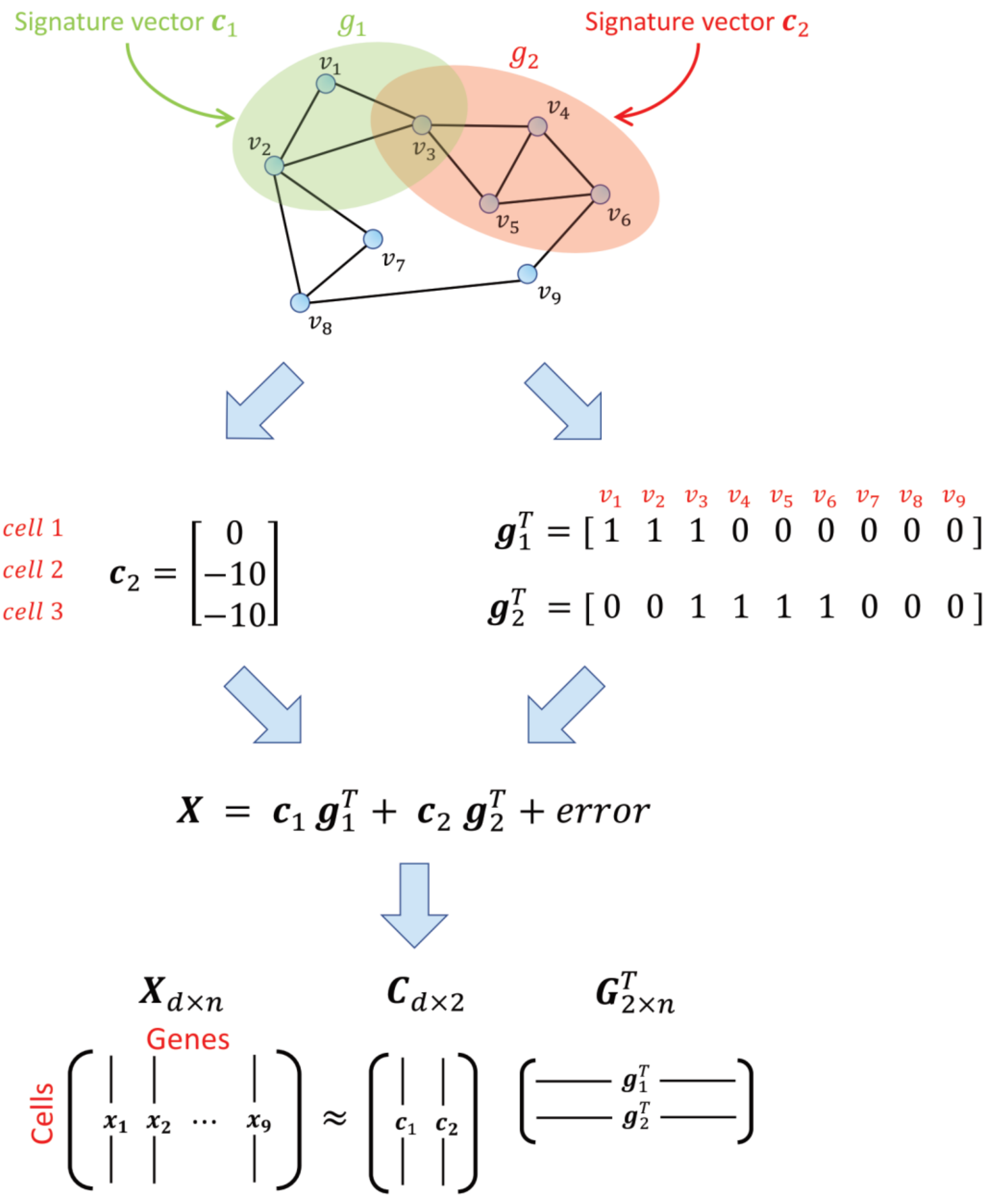

Supplementary Figure 10 CG Decomposition Overview

The first procedure is called GreedyCG and the second procedure is called FindSubgraph in the description below. FindSubgraph is called as a subroutine in GreedyCG. In the following we describe each algorithm separately.

Supplementary Figure 11 shows GreedyCG algorithm in pseudocode. The number of seed nodes specifies how many times the algorithm calls the FindSubgraph procedure to find each subnetwork. We provide more details about seed nodes when describing FindSubgraph algorithm (Supplementary Figure 12).

The GreedyCG algorithm starts by setting the residual matrix X_{res} to the original expression matrix. Then the algorithm consecutively solves rank-1 approximations of the data matrix and subtracts them from the residual matrix. Each rank-1 solution identifies a connected subnetwork and a signature expression vector. In each iteration, the algorithm constructs one row of G which is the binary indicator vector of the detected subnetwork. The algorithm repeats this procedure r times to find a rank-r factorization. Finally, it finds the optimal C via least squares. Signature vectors of the detected subnetworks do not need to be saved while constructing rank-1 solutions because C is unconstrained and so can be computed optimally and efficiently for a given G by least-squares.

Each subnetwork detection iteration involves an inner loop. The inner loop finds s candidate solutions by calling the FindSubgraph algorithm s times, each time with a different seed node. For each seed it selects the solution that minimizes the approximation of the current residual data. This step is added since our subnetwork detection is a heuristic and depends on the initial seed. Increasing s will increase the chance of finding a rank-1 solution that better approximates the residual expression data.

Algorithm 1: `GreedyCG`

```
Input: X ∈ ℝ^{m×n}, M(V, E)
Output: C ∈ ℝ^{m×r}, G ∈ {0, 1}^{r×n}
Parameters: number of subnetworks r, number of seeds s

X_res = X
for k = 1 to r do
    seeds = top s nodes with largest column norm in X_res
    for v in seeds do
        c, g = FindSubgraph(X_res, M, v)
        Candidate[v] = (c, g)
        CandidateError[v] = ||X − cg^T||_F^2
    v* = argmin(CandidateError)
    c, g = Candidate[v*]
    X_res = X_res − cg^T
    G[k, :] = g^T
C = argmin_{C*} ||X − C*G||_F^2
return C,G
```

Supplementary Figure 11

`FindSubgraph` uses a greedy approach to find each subnetwork. The algorithm creates a subnetwork that initially only contains the given seed node. Then the algorithm expands the subnetwork as follows: In each iteration, it considers all the neighboring nodes that are connected to the current selected subnetwork and computes how much the addition of each neighboring node would improve the approximation error. It adds the node that causes the maximum improvement to the subnetwork and repeats this procedure until the subnetwork reaches the maximum size given by parameter l, or until none of the neighboring nodes will improve the approximation error.

The BindaryInicator() function used in the pseudocode is a function that maps a set of nodes to a binary vector of size n in which the positions that correspond to the nodes in the given set are set to 1. Also note that in the case of rank-1 factorization the optimal c for a given g found by least-squares is $\frac{1}{|g|}Xg$.

Algorithm 2: `FindSubgraph`

Input: $X \in \mathbb{R}^{m \times n}$, $M(V, E)$, node $v \in V$
Output: $\mathbf{c} \in \mathbb{R}^{m}$, $\mathbf{g} \in \{0, 1\}^{n}$
Parameters: maximum size l

$S = [v]$
error = $||X||_F^2 - ||\mathbf{x}_v||_2^2$
while $|S| < l$ **do**
  for *node p in neighbors of S* **do**
    $S' = S \cup p$
    g= *BinaryIndicator*(S')
    $\mathbf{c} = \frac{1}{|\mathbf{g}|} X\mathbf{g}$
    NewError[p] = $||X - \mathbf{c}\mathbf{g}^T||_F^2$
  p* = *argmin*(NewError)
  if *NewError[p*] <error* **then**
    $S = S \cup p^*$
    error = NewError[p*]
  else
    break loop
g= *BinaryIndicator*(S)
$\mathbf{c} = \frac{1}{|\mathbf{g}|} X\mathbf{g}$
return c,**g**

Supplementary Figure 12

Given the above description of the algorithm, there are some computational optimizations possible. In particular we can run the subnetwork expansion part in algorithm 2 more efficiently. This part of the algorithm seeks to answer the question: "given a partial subnetwork expressed as a binary indicator vector g and a set of neighbors of g, which node p should be added to make $g' = g \cup p$ to improve the approximation error as much as possible?"

In other words, it looks for g' that maximizes:

$$||X - cg^T||_F^2 - ||X - c'g'^T||_F^2$$

By expressing c as the average of the columns in X defined by g, and rewriting the Frobenius norm using the trace operator we can write the first term in the above equation as: $Tr(X^TX) - \frac{1}{|g|} g^T X^T X g$.

Writing the second term in a similar form and assuming that $|g| = k$, we can rewrite our goal as finding node p that forms $g' = g \cup p$ such the following is maximized:

$$\frac{1}{k+1} g'^T X^T X g' - \frac{1}{k} g^T X^T X g$$

Therefore, we can get a significant speedup in the implementation of the inner loop in algorithm 2 by computing $X^T X$ once and finding the gain obtained after adding each neighbor node p to g by only computing additional sums over the necessary elements of $X^T X$.
